# Automated Detection of Malarial Retinopathy in Digital Fundus Images for Improved Diagnosis in Malawian Children with Clinically Defined Cerebral Malaria

**DOI:** 10.1038/srep42703

**Published:** 2017-02-15

**Authors:** Vinayak Joshi, Carla Agurto, Simon Barriga, Sheila Nemeth, Peter Soliz, Ian J. MacCormick, Susan Lewallen, Terrie E. Taylor, Simon P. Harding

**Affiliations:** 1 VisionQuest Biomedical LLC, Albuquerque, NM, United States; 2 Eye and Vision Science, Institute of Ageing and Chronic Disease, University of Liverpool, Liverpool, United Kingdom; 3 Malawi-Liverpool-Wellcome Trust Clinical Research Programme, Blantyre, Malawi; 4 Centre for Clinical Brain Sciences, University of Edinburgh, United Kingdom; 5 Kilimanjaro Centre for Community Ophthalmology, University Cape Town Groot Schuur Hospital, Cape Town, South Africa; 6 Department of Osteopathic Medical Specialties, College of Osteopathic Medical Specialties, Michigan State University, East Lansing, MI, United States; 7 Blantyre Malaria Project, Blantyre, Malawi

## Abstract

Cerebral malaria (CM), a complication of malaria infection, is the cause of the majority of malaria-associated deaths in African children. The standard clinical case definition for CM misclassifies ~25% of patients, but when malarial retinopathy (MR) is added to the clinical case definition, the specificity improves from 61% to 95%. Ocular fundoscopy requires expensive equipment and technical expertise not often available in malaria endemic settings, so we developed an automated software system to analyze retinal color images for MR lesions: retinal whitening, vessel discoloration, and white-centered hemorrhages. The individual lesion detection algorithms were combined using a partial least square classifier to determine the presence or absence of MR. We used a retrospective retinal image dataset of 86 pediatric patients with clinically defined CM (70 with MR and 16 without) to evaluate the algorithm performance. Our goal was to reduce the false positive rate of CM diagnosis, and so the algorithms were tuned at high specificity. This yielded sensitivity/specificity of 95%/100% for the detection of MR overall, and 65%/94% for retinal whitening, 62%/100% for vessel discoloration, and 73%/96% for hemorrhages. This automated system for detecting MR using retinal color images has the potential to improve the accuracy of CM diagnosis.

Cerebral malaria (CM) is an often-fatal clinical complication associated with *Plasmodium falciparum (Pf*) malaria infection. The 2015 World Malaria Report estimates that malaria affected over 214 million people, and claimed the lives of 438,000 people worldwide, about 67% of whom were African children under 5 years of age; the majority of these pediatric deaths was attributed to CM^1^. Annually, CM results in the loss of 35.4 million disability adjusted life years due to mortality and morbidity of clinically diagnosed CM patients in Africa^1^,^2^. The standard WHO clinical case definition of CM (physical symptoms such as seizures, convulsions; Blantyre coma score <2, detection of *Pf* parasites in the peripheral blood, and exclusion of other causes of coma (e.g. meningitis, hypoglycemia) refs 3,4); may misclassify up to 25% of cases^5^. Because asymptomatic *Pf* infection is common parasitemia in malaria-endemic areas, CM is often over-diagnosed. The WHO clinical case definition overlooks other non-malarial illnesses with similar clinical symptoms “masquerading” as CM. The result is a significant false positive rate (FPR) for CM which leads to inaccurate epidemiological estimates of disease incidence, incorrect classifications in research and clinical settings, and potentially inadequate treatment of the underlying illness^6^,^7^,^8^. If non-malarial illnesses with incidental parasitaemia go undiagnosed and thus improperly treated, potentially avoidable death or neurologic disabilities may result. In summary, though the WHO criteria are highly *sensitive* to the presence of CM, they generate a significant proportion of false positives due to misclassification resulting from individuals with asymptomatic, incidental *Pf* parasitemia^5^.

Lewallen *et al*.^8^ first reported on retinal lesions associated with CM, and with others subsequently coined the term malarial retinopathy (MR) to describe the characteristic features: retinal whitening, vessel discoloration, and white-centered hemorrhages^4^,^9^,^10^. In an autopsy-based study, comatose children who tested positive for *Pf* (thus satisfying the WHO criteria), the presence of any feature of retinopathy (hemorrhage or whitening or vessel discoloration) had a high sensitivity and specificity for the pathological hallmark of CM, i.e. sequestration of parasitized red cells in the cerebral microvasculature^5^. Non-malarial causes of coma and death were identified in all comatose, parasitemic children with normal ocular funduscopic examinations. CM results in the appearance of retinal lesions such as retinal whitening and vessel discoloration that are visible in color retinal images, and reflect disease processes occurring in the brain^11^,^12^,^13^. Adding a clinical test for MR to the current WHO diagnostic protocol for CM may improve the specificity of CM diagnosis from 61% (WHO criteria alone) to greater than 95% (WHO criteria + MR detection)^5^,^9^,^14^. Thus, retinal screening for MR has potential to improve the specificity of the standard clinical case diagnosis of CM and reduce false positive rate. Apart from the clinical utility, MR detection may assist in increasing the accuracy of CM epidemiology, studies in CM pathogenesis, and reducing sample heterogeneity in studies of mechanisms and new treatments that may lead to better care.

As stated earlier, the WHO criteria are sufficiently sensitive and effective in detecting true CM patients, but may fail in detecting CM-negative cases giving rise to false positives. The objective of MR detection is to prevent such false positive diagnoses of CM, as illustrated in Fig. 1a. The proposed MR detection system will not replace the current CM diagnostic protocols (WHO criteria), but instead will supplement the current protocols to increase the specificity of CM diagnosis. The specificity of WHO criteria is reported as 61% by Beare *et al*.^14^, and up to 77% by Taylor *et al*.^5^; which can result into an FPR of up to 23%, or in other words, at least 23% of the admitted patients (with non-CM diseases) getting unnecessary treatment for CM and not being treated for the real cause. Once CM is diagnosed clinically, investigation of other diseases is ignored. Thus, a severe non-malarial illness causing coma, and detected with an asymptomatic *Pf* parasite, is often misdiagnosed as CM, which can result in incorrect treatment and potentially avoidable death. Therefore, it is of utmost importance to use MR detection test with high specificity *in addition* to the current WHO diagnostic protocol for CM, to triage patients with non-CM diseases which can be investigated for other causes of coma, such as other most prevalent diseases in the geographical region. Our proposed MR detection system with a goal FPR of 5%, when added to the current clinical protocol (WHO criteria), may achieve an estimated overall FPR of 1.15%, and increase the specificity of CM diagnosis.

Figure 1b presents a model for CM diagnosis and clinical management that shows the advantage of including MR detection. CM is currently diagnosed based on WHO criteria and according to the current clinical protocols in Africa, as soon as the clinical diagnosis of CM is made, the patient is treated with an antimalarial medication (Artesunate or Quinine) irrespective of whether MR is detected or not^15^. An important point to make is that although the current clinical diagnosis of CM (WHO criteria) has low specificity, it *is highly sensitive* (>95%). The utility of MR detection is in reducing the false positive rate and increasing the positive predictive value (PPV). Therefore, the sensitivity of MR detection test may be traded off up to some extent to improve specificity, when this test is used in conjunction with the current WHO protocols for CM diagnosis; as both MR-positive (that indicates true CM) and MR-negative (that indicates non-CM disease) patients get treated for CM with anti-malarial (Fig. 1b). The patients meeting the WHO criteria who have no detectable MR will still receive antimalarial treatment for their *Pf* infection; but the absence of MR should prompt clinicians to continue looking for alternative causes of coma in these patients.

The presence of MR is currently detected by dilated eye exams using binocular indirect ophthalmoscopy (BIO), or analysis of retinal fundus images by an ophthalmologist^4^,^16^. However, at present, the lack of specialized and expensive equipment and expertise required for BIO in most malaria-endemic areas remains a barrier to wider use of MR detection in clinical practice^15^,^17^,^18^,^19^. Utilizing retinal color images provides another option; but scarcity of ophthalmic experts to interpret them presents another challenge. In this context, a fast, accurate, and fully automated software system for MR detection using retinal color images could provide quick diagnostic assistance and improve care.

As reported previously in the literature, Joshi *et al*. used an automated method for MR hemorrhage detection using retinal color images^20^. Zhao *et al*. used graph methods and saliency maps to detect vascular leakage (sometimes associated with the whitening seen in MR) in fluorescein angiogram images^21^, and also described an automated method for detecting intravascular filling defects (the basis for vessel color changes in MR) using fluorescein angiogram images^22^.

We developed and tested a fully automated method using retinal color images that detects MR lesions and classifies retinal images into those with or without MR, using a classification algorithm based on combinations of detected MR lesions. The article is organized as follows: Section 2 describes the methods and materials required for this research. Results are shown and discussed in Section 3. Finally, the discussion and conclusion are presented in Section 4.

## Methods

### Regulatory approval

The retrospective data utilized and experiments performed as part of the proposed software development conformed to international research standards and received institutional review board (IRB) approval from the *Ethical and Independent Review Services* (E&I study #14119-01A and 16093-01) and from the research ethics committees at Michigan State University, the University of Liverpool, and the University of Malawi College of Medicine.

### Dataset

A dataset of retinal color images was generated from patients who met WHO criteria for CM and who had been admitted to an ongoing study of malaria pathogenesis. The parent dataset consists of N = 176 patients containing 16 controls with normal ocular funduscopic exams (per the experienced ophthalmologists) and 160 cases with WHO-defined CM who had signs of MR detected by a trained ophthalmologist. All of the available control images (N = 16) were included in our study data as only a limited number of controls were admitted. In order to match with the proportion of controls (~20%) and cases with MR (~80%) in the general population in malaria endemic areas, we selected N = 70 cases with MR randomly, out of the available N = 160 cases, the only constraint being that the retinal images have adequate quality for algorithm processing. Therefore, the dataset utilized for algorithm development consists of N = 86 patient’s images with N = 70 who had signs of MR and N = 16 controls. Of the 70 images with MR, 67 images had retinal whitening, 57 had white-centered hemorrhages, 49 had vessel discoloration, and 53 images had more than one MR lesion. The images were collected between 2006 and 2014 from patients admitted to the Pediatric Research Ward at the Queen Elizabeth Central Hospital (QECH), in Blantyre, Malawi. The images were captured using a Topcon 50-EX retinal camera with a field of view (FOV) of 50 degrees. All images were anonymized by the Liverpool Ophthalmic Reading Center and provided under a numeric identifier. The Topcon 50-EX camera provides a limited FOV of the retina and hence it does not capture the necessary region of the retinal posterior pole in a single image. Therefore, our collaborators at the University of Liverpool stitched multiple images of a patient to create a single mosaic image (Fig. 2a), using i2K Retina^®^ software (DualAlign™ LLC, New York, USA).

A retinal grader (SN), trained at Liverpool Ophthalmic Reading Center, annotated and graded the 86 mosaic images. The detailed annotations used a color-coding scheme to denote different MR lesions. Figure 2a,b show the retinal image mosaic and its ground truth annotation, where retinal whitening is annotated in blue, vessel discoloration in green, and hemorrhages in red. The annotations were validated by an ophthalmologist with expertise in MR (SL). The annotated mosaic database was used to develop algorithms to detect the three retinal signs of MR, as described in the following sections.

### Algorithm development

Algorithms were developed to detect each of the three MR abnormalities and determine the presence or absence of MR. The following sections describe the image processing methodologies for detection of each abnormality.

#### Vessel discoloration

Retinal vessel discoloration is a feature uniquely associated with MR. Vessel discoloration presents as discoloration from red to orange or white^8^. Figure 3a shows an example of vessels presenting with discoloration due to CM. Figure 3b presents the grader’s annotation of that image. The image processing flow for vessel discoloration detection is described below.

- Vessel segmentation and pre-processing: The first step in detecting vessel discoloration is to segment the retinal vasculature. VisionQuest’s vessel segmentation algorithm^23^ was adapted by incorporating information from green channel of RGB color space (Fig. 4a) and ‘a’ channel of the CIE-Lab color space (Fig. 4b) that represents a green-red component of the vessel pixels. The ‘a’ channel was helpful in segmenting small, subtle vessels and especially the discolored vessels which could not be segmented accurately using VisionQuest’s segmentation algorithm based on green channel features. During the analysis of the ground truth annotations, it was observed that the wider vessels close to the optic disc do not present with discoloration, and hence they were used as an indicator of normal vessel color. This information was used to normalize the vessel color within each image by automatically identifying the dark wider vessels closer to the optic disc, using contrast enhancement and intensity thresholding. For each color channel, the mean value was calculated for all normal vessel pixels. The algorithm then identified the discolored vessels by calculating the intensity difference between each pixel of the remaining vasculature and the mean intensity value of normal vasculature.
- Feature extraction and classification: We based our feature extraction on three factors: 1) color of the discolored vessels is orange or white, 2) edge of the wall of discolored vessels has low contrast, and 3) regions close to the vessel wall get discolored first when the pathology appears. Based on these considerations, statistical image features (median, standard deviation, kurtosis, skewness, moment), and 10^th^, 25^th^, 50^th^, 75^th^, 95^th^ percentile of the content in a vessel segment were extracted from various color channels (RGB, HSL, CIE-XYZ, CMYK, Luv, Lch) in the center and wall areas of the vessels. We also calculated the gradient of ‘red’, ‘green’ and ‘a’ channels to measure the contrast of a vessel.
- The feature set consists of information from 22 different image representations (19 color channels and 3 gradients) each in terms of 10 statistical features, extracted from inner and outer parts of vessel segments; yielding the total features set of 22 × 10 × 2 = 440 features. During the training phase, ANOVA was used to determine which of the features were the most significant (p < 0.05) and thus reduced the feature space by 75%. The features were normalized to have mean ‘zero’ and ‘unit’ standard deviation. A leave-one-out partial least squares (PLS) classifier was utilized to classify the vessel segments using 5-fold cross-validation. Figure 5b shows a retinal image with automated detection of discolored vessels, marked in blue.

#### White-centered hemorrhages

Retinal hemorrhages of MR are predominately white-centered, intra-retinal and blot like hemorrhages, which most commonly involve the inner retinal layers. They can occur in isolation, in small groups, or in large clusters. Figure 6a shows examples of MR hemorrhages and Fig. 6b shows the grader’s annotation.

- Hybrid method: A hybrid method for detection of hemorrhages was developed that combined a supervised classification approach previously reported by the author^20^, and a newly developed method for unsupervised detection of MR hemorrhages. The details of the supervised classification method are provided elsewhere^20^. Briefly, this method used a feature extraction and classification of image watershed regions called splats. A set of 43 image features consisting of color, difference of Gaussians, and local contrast, was extracted from each splat. A linear k-Nearest Neighbors (kNN) classifier was trained using the splat features, which then calculated a probability (0–1) of each splat in test images whether it belongs to a hemorrhage (Fig. 7a).
- The unsupervised detection approach utilized color features from various color channels: ‘a’ (Lab), ‘u’ (Luv), ‘Q’ (YIQ), ‘C’ (LCH). The information from these four color channels was combined using a pixel-based image multiplication operation where each output pixel’s value depends on the multiplication of corresponding input pixel values^24^,^25^. This step improved the contrast of hemorrhage-lesions relative to a retinal background. A watershed segmentation was then used, where the contrast-improved image determined the gradient magnitude and formed the watershed region boundaries. The resulting hemorrhage segmentation image assigned a probability to each watershed region of being inside a hemorrhage (Fig. 7b).
- The unsupervised approach could detect well the small/subtle hemorrhages otherwise missed by the supervised classification method; whereas, the supervised method was efficient in segmenting the large hemorrhages. The results of hemorrhage detection from supervised and unsupervised methods were combined to form a hybrid image (Fig. 7c). In order to minimize the number of false positive detections, the morphological features of each probable hemorrhage candidate were utilized, which include eccentricity, solidity, and bounding box.

#### Retinal whitening

Retinal whitening is thought to be a manifestation of hypoxia, and it has been postulated that it is the result of oncotic cell swelling of neurons in response to hypoxic stress^13^. The severity and pattern of whitening in MR is thought to be unique to CM^17^ (Fig. 8a). The proposed method for whitening detection combines pre-processing techniques to remove camera reflex that decreases the number of false positives, and feature extraction and classification to detect the presence of whitening.

- Preprocessing for reflex minimization: The first step in the detection of whitening is to minimize imaging artifacts such as reflex from the internal limiting membrane that often results in false positive detection. A reflex is often confused with true whitening and it is a challenging task to differentiate between the two, as the color features of whitening largely overlap with that of the reflex. As shown in Fig. 8a, true whitening is creamy or fuzzy white in color, whereas reflex presents with shiny and bright white color. Figure 8b shows the grader’s annotation of retinal image in Fig. 8a, with true whitening marked in blue and reflex marked in shiny green color. To minimize the effect of reflex, the color channels: green (RGB), k (CMYK color space), X and Z (CIE-XYZ color space), and ‘L’ (HSL color space), were used. These color channels provide a significant distinction between a true whitening region and reflex. The information from these five color channels was combined using a pixel-based image multiplication operation^24^,^25^, which improved the contrast between true whitening and reflex. A white top-hat transform was then applied to the contrast-improved image, which highlighted the reflex and separated it from true whitening (top-hat transform image).
- Apart from the color features, the other distinguishing feature between whitening and reflex is the smooth and fuzzy texture of whitening versus the sharp texture of reflex with strong edges, which are also used by a grader in differentiating the whitening from reflex. This difference was detected using textural filter functions such as entropy, range, and standard deviation^26^, applied to the top-hat transformed image. The resulting texture-filtered image was further enhanced using contrast equalization. The contrast-equalized image that highlighted the reflex region, was subtracted from the original image (Fig. 9a) using a pixel-based image subtraction operation, and post-processed for illumination normalization, Gaussian smoothing, and noise removal. The final outcome of this process minimized the reflex and preserved the true whitening (Fig. 9b).
- Feature extraction and classification: A marker controlled watershed segmentation was applied to the green channel of the preprocessed image (Fig. 9b), which divides the image into number of watershed regions called splats based on region homogeneity (image regions with similar intensity pixels) as shown in Fig. 10b 27. This process uses gradient magnitudes of the green channel at multiple scales and utilizes their maximum for segmentation^28^. The watershed segmentation of the gradient magnitude image is performed, which generates the splats. Multiple color channels of the processed image, such as ‘k’ (CMYK color space), green (RGB color space), and ‘X and Z’ (CIE-XYZ color space) were used to extract features from each of the splats. The feature set included: 1) Haralick textural features such as contrast, correlation, energy, homogeneity, entropy, dissimilarity, and other textural features obtained from a gray-level co-occurrence matrix (22 features)^29^,^30^,^31^; 2) Statistical features: median, standard deviation, kurtosis, skewness, moments, and percentile at various values (10 features); 3) Morphological features: Eccentricity, Solidity, Bounding box. The feature set included a total of 131 features. The entire feature set was utilized for classifier training and testing. The features were normalized to have ‘zero’ mean and ‘unit’ standard deviation. A partial least square (PLS) classifier was trained to learn the properties of whitening, and assign each splat in the test images a probability of being inside the whitening region (Fig. 11c). This probability map can be thresholded at various values to detect whitening with different sensitivity/specificity characteristics. Similarly, as utilized for the hemorrhage detection, the morphological features such as size, eccentricity, solidity, and bounding box, were used to minimize false positive detections of whitening.

#### Combination of individual algorithms for MR detection

The ultimate goal of MR detection software system is to reduce the false positive rate (FPR) by accurately identifying pediatric patients for the presence or absence of retinal signs of MR (MR/no-MR). Since anti-malarial treatment will be given to all clinically diagnosed pediatric CM patients (regardless of MR status), specificity was prioritized over sensitivity in the algorithm. Hence, we tuned individual MR abnormality detection algorithms to high specificity settings. The MR detection system combined the outputs of the detection algorithms for hemorrhages, vessel discoloration, and retinal whitening, using PLS classifier. The classifier performance was calculated using leave-one-out cross-validation technique against the ground truth provided by VisionQuest’s expert grader and validated by an ophthalmologist (SL).

#### Analysis

For each of the three MR lesions, the algorithm performance was calculated in terms of accuracy of image-based classification. For each image, the probability map obtained for detection of a lesion was converted to a binary image using various thresholds and was compared against the annotated ground truth image. The image-based classification analysis determined the algorithm performance in classifying each retinal image as that with or without a lesion. This classification technique considers a given image as positive detection, if at least one of the lesions annotated in the ground truth is detected by the algorithm at a given threshold. The image-based sensitivity is defined as the fraction of images marked positive in the ground truth, that are detected as positive by the algorithm; while the image-based specificity is defined as the fraction of images marked negative in the ground truth, that are detected as negative by the algorithm. For the image-based classification analysis, a receiver operating characteristic (ROC) curve was determined. Since the aim of MR detection algorithms is to reduce an FPR in CM diagnosis and achieve high specificity, we tuned each algorithm to a high specificity setting (>90%).

## Results

The dataset of N = 86 images (Refer to Dataset details) was utilized for testing each algorithm using leave-one-out methodology. The algorithm performance is demonstrated below.

### Vessel discoloration

The dataset contained 49 images with vessel discoloration and 37 without discoloration. In order to calculate the performance for classifying each image as with or without vessel discoloration, the number of vessel segments classified as containing discoloration were counted in the given image and the count was used to determine if the image contains discolored vessels. The image-based classification obtained an area under the curve (AUC) of 0.85 with specificity of 100% and sensitivity of 62% (Fig. 12a). The algorithm can also be operated at high sensitivity point of 90% at specificity of 66%.

### Retinal hemorrhages

The dataset had a distribution of 57 images with hemorrhages and 29 images without hemorrhages. The average number of hemorrhages per image was 15. In order to classify each image as with or without hemorrhages, the number of hemorrhages detected by algorithm were counted in the given image and the count was used to determine if the image contains hemorrhages. The classification of images with or without hemorrhages achieved an AUC of 0.89, with specificity of 96% and sensitivity of 73% (Fig. 12b). The algorithm can also be operated at high sensitivity point of 100% at specificity of 78%.

### Retinal whitening

In the given dataset, whitening was present in 67 images and 19 images presented with no whitening. With the ultimate goal of determining the presence or absence of whitening in a given image, three features were extracted from each image, i.e. 1) Maximum probability of whitening assigned to a splat in the image, 2) Sum of non-zero probabilities assigned to splats, 3) Product of non-zero probabilities assigned to splats. The PLS classifier was used to classify each image as that with or without the whitening, based on the above image-based features. The classification of images with or without whitening achieved an AUC of 0.81, with specificity of 94% and sensitivity of 65% (Fig. 12c). The algorithm can also be operated at high sensitivity point of 78% at specificity of 65%.

### MR detection

The individual MR lesion detection algorithms were combined using a PLS classifier for detecting the presence or absence of MR with high specificity, while maintaining a minimum sensitivity of 90% for MR detection. The MR detection algorithm yielded an AUC of 0.97 with specificity of 100% at sensitivity of 95%. The positive predictive value (PPV) for MR detection was 0.98. PPV was not calculated for individual MR lesions due to unavailability of prevalence data per lesion. Table 1 shows the performance of detection algorithms for vessel discoloration, hemorrhages, retinal whitening, and MR detection; in terms of AUC, and tuned at high specificity while maintaining moderate sensitivities.

## Discussion

Our results suggest that automated software has potential to be used to detect MR, obviating the need for bedside ophthalmoscopy. If this is feasible in malaria endemic areas, it would represent simplified approach for improving the diagnosis of CM in patients who satisfy the established WHO definition of CM: coma, peripheral parasitaemia, and no other obvious cause of coma. The AUCs for individual MR lesion detection in the range of 0.81–0.89 indicate the capability of respective algorithms in distinguishing between an MR abnormality and retinal background. The AUC of 0.97 for MR detection overall suggests that, given a random retinal image of a pediatric patient with clinically diagnosed CM, the proposed MR detection system will correctly identify presence or absence of MR in 97% of the patients. This system, operating at a high specificity setting for diagnosis of MR (100% specificity and 95% sensitivity) could represent a potentially significant reduction in the false positive rate in the diagnosis of cerebral malaria.

**Figure 1:**
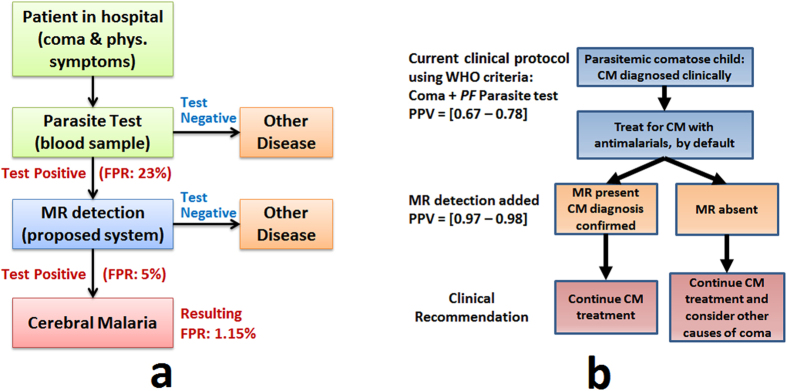
(**a**) Proposed CM diagnosis using MR detection, (**b**) CM clinical management using MR detection.

**Figure 2:**
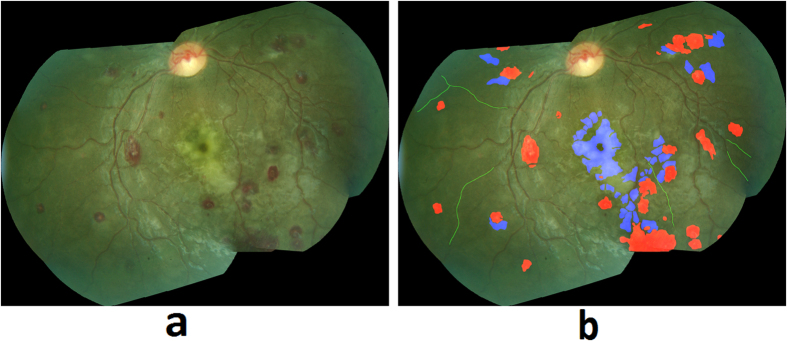
(**a**) Retinal image mosaic, (**b**) Grader’s annotation of MR lesions.

**Figure 3:**
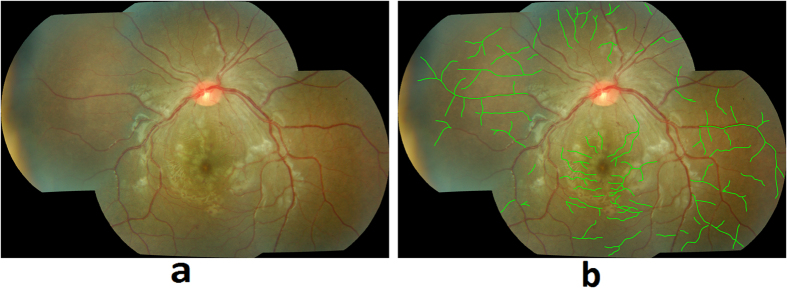
(**a**) Vessel discoloration, (**b**) ground truth annotation.

**Figure 4:**
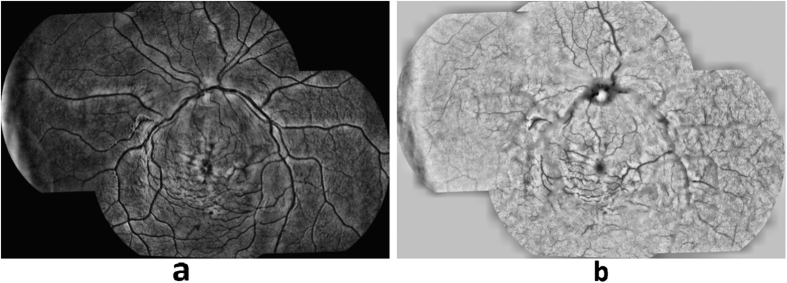
(**a**) Enhanced green channel, (**b**) Enhanced ‘a’ channel.

**Figure 5:**
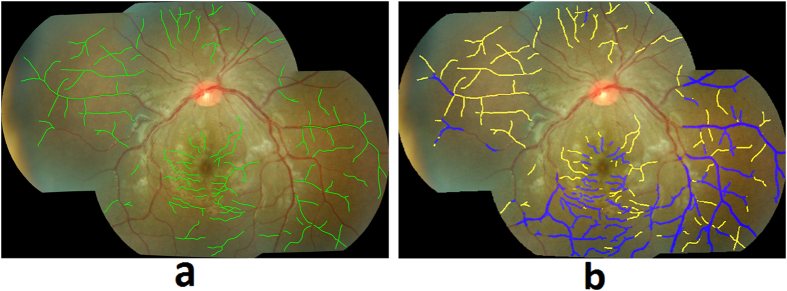
Image showing: (**a**) discolored vessels (green) annotated by grader, (**b**) discolored vessels detected (blue) or missed (yellow) by the algorithm.

**Figure 6:**
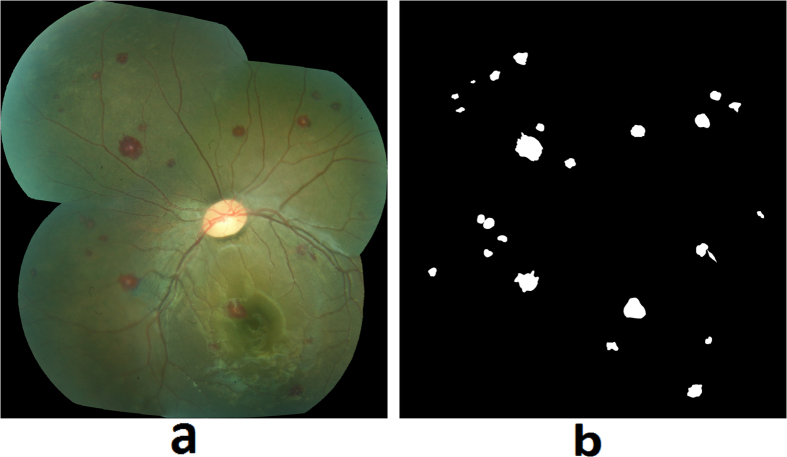
MR hemorrhages: (**a**) Fundus image, (**b**) Grader’s annotation.

**Figure 7:**
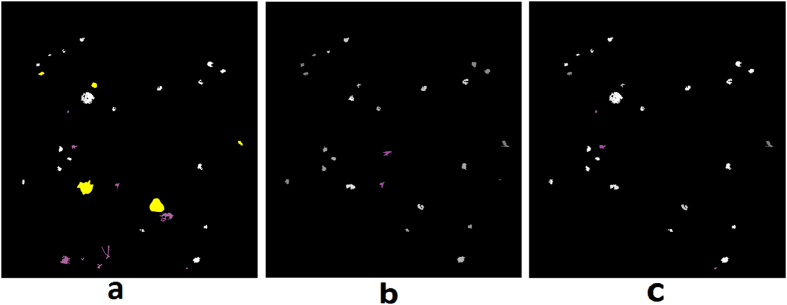
Hemorrhage detection using: (**a**) Supervised classification, (**b**) Unsupervised classification, (**c**) Hybrid method. Each image shows false positive (magenta) and false negative (yellow) detections.

**Figure 8:**
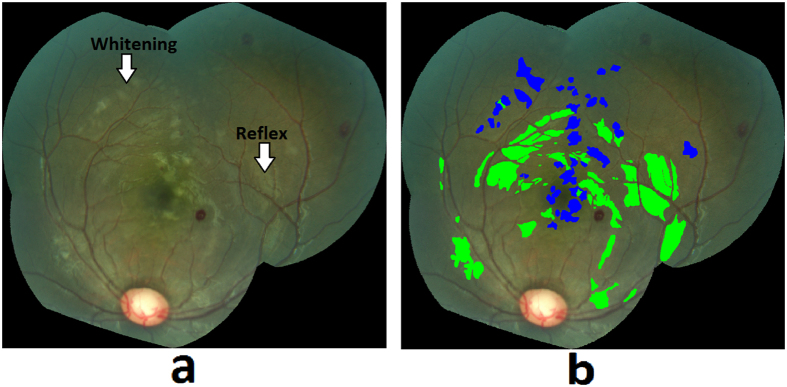
(**a**) Retinal whitening and camera reflex, (**b**) Grader’s annotation for whitening in blue and reflex in shiny green color.

**Figure 9:**
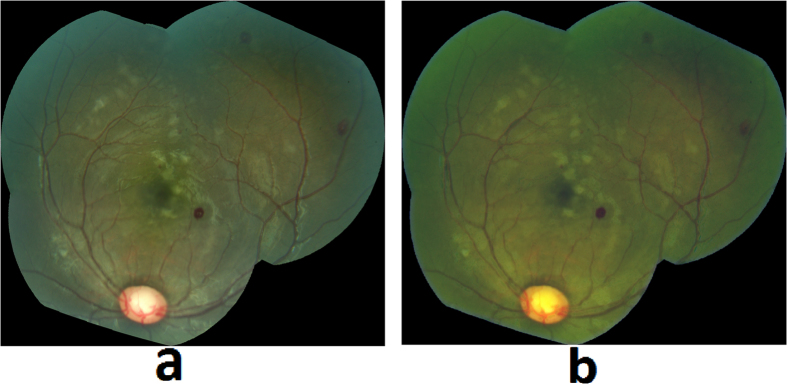
(**a**) Retinal image, (**b**) Preprocessed image with reflex minimization.

**Figure 10:**
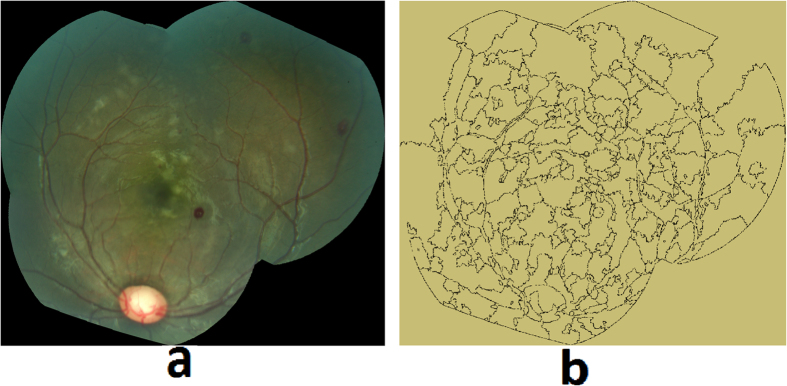
(**a**) Retinal image, (**b**) Image splats (regions) formed by watershed segmentation.

**Figure 11:**
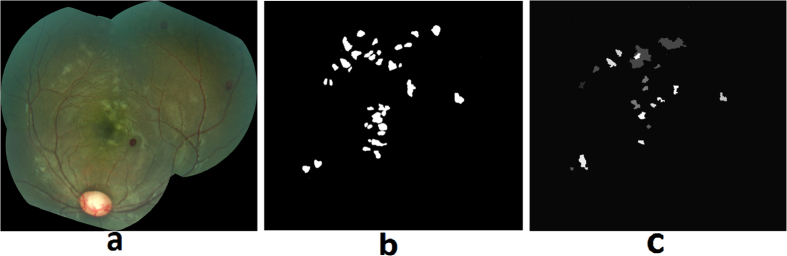
MR whitening detection: (**a**) Retinal image, (**b**) Grader’s annotation of whitening, (**c**) Image splats with assigned probability of whitening.

**Figure 12:**
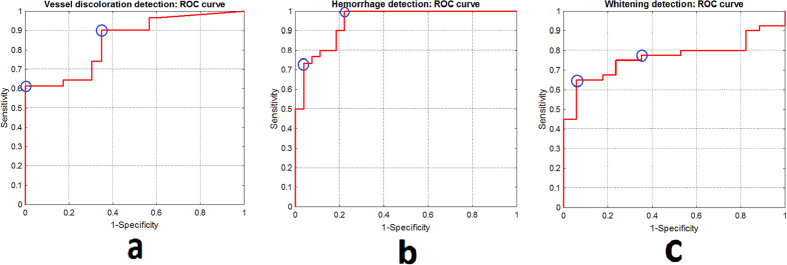
ROC curves for: (**a**) vessel discoloration detection, (**b**) hemorrhage detection, (**c**) whitening detection.

Using the specificity value in the range of 61–77% of WHO criteria for the diagnosis of histologically confirmed CM^5^,^14^, and a CM prevalence of 46% among patients who are comatose and parasitemic^32^, we calculate the current positive predictive value (PPV) for CM detection to be in the range of 0.67–0.78 (Refer Fig. 1b). When the standard WHO clinical case definition is supplemented with the proposed MR detection test, it results in PPV in the range of 0.97–0.98.

**Figure 13:**
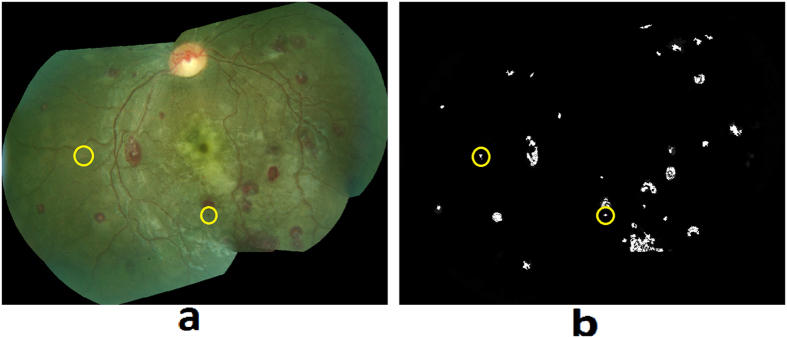
(**a**) Retinal image, (**b**) Automated hemorrhage detection.

Apart from its ultimate goal of improving the diagnostic accuracy of CM, the automated algorithms may also assist in the observation and detection of subtle or small lesions in retinal images collected sequentially over time, which may be indicative of regression of MR due to a patient recovering from CM. Such regressing lesions may be clinically significant for making number of prognostic observations such as: 1) indication of a regressing disease, 2) disease timeline, 3) effect of a therapy, 4) occurrence or regression of lesions in relation with other clinical symptoms, 5) location of regressing lesions in relation with location of ischemia or retinal hypoxia. Figure 13a shows a retinal image with two subtle hemorrhages (marked in yellow circle) detected by the algorithm (Fig. 13b), which were confirmed as the signs of a regressing disease by an ophthalmologist that analyzed the retinal images. The algorithm-based detection and comparison of lesions for a longitudinally collected retinal image data may be helpful in quantifying and recording the lesion regression rate, prevalence, severity, location; the information that may assist a physician.

The proposed method has a few limitations. First, several imaging and image processing artifacts reduce the accuracy of MR lesion detection. A commonly observed artifact is a light reflex from the internal limiting membrane, which results in false positive detection for lesions such as retinal whitening. As discussed earlier, the retinal images can be preprocessed to minimize the effect of reflex by utilizing various color-spaces and advanced textural feature extraction methods such as deep learning^33^,^34^; which are capable of extracting multiple levels of representation and abstraction that can help differentiate between true whitening and reflex.

**Figure 14:**
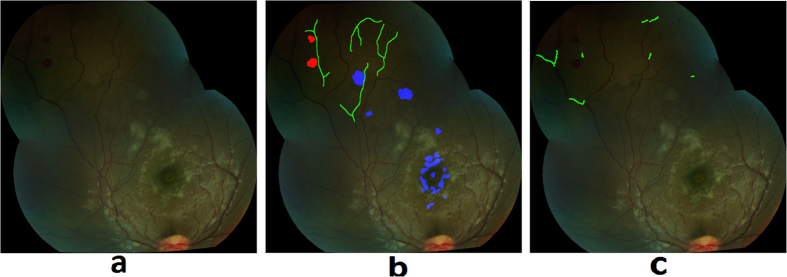
(**a**) Retinal image, (**b**) Ground truth annotation, (**c**) Vessel discoloration detection.

Other factors contributing to the reduction in accuracy are non-uniform illumination across a retinal image, known as vignetting^35^, and low contrast. These artifacts cause the algorithms to miss MR lesions such as hemorrhages and vessel discoloration, resulting in false negative detection of these lesions. Figure 14a,b show an example of a non-uniformly illuminated retinal image with low contrast in some of the regions, and ground truth annotation for MR lesions, respectively. Figure 14c shows the algorithm-based detection of only a few discolored vessel fragments relative to the ground truth (low sensitivity), as a result of low illumination in the region of interest. Since the count of algorithm-detected discolored vessels in the given image is low, this image is classified as “not containing vessel discoloration” by the algorithm. Furthermore, both of the hemorrhages in this image could not be detected by the algorithm. Since the proposed algorithms use color and intensity information for detection of MR lesions, it is important to normalize the retinal images and/or improve contrast before the extraction of lesion features. The illumination artifact can be corrected by using several methods previously reported for intensity and color normalization^36^.

The retinal images used in the algorithm development were obtained using a table-mounted retinal camera Topcon 50-EX, operated by a trained ophthalmologist in a research setting. Table-mounted cameras provide good image quality which is advantageous to the software-based analysis of images. However, these cameras are not easily accessible and affordable to many clinics in Africa where CM is most prevalent. Portable or cellphone based retinal cameras are more accessible options and may prove useful in the future for image acquisition. To date image quality in these systems has only proved moderate at best and worse in the hands of a medical technician or nurse rather than an ophthalmologist^37^. Therefore, it will be necessary to test, modify, and validate the proposed algorithms on commonly available image data obtained in clinical settings in Africa.

Finally, the training and test datasets used in the proposed algorithm development were selected based on availability of MR and control images. Only a limited number of control images (pediatric patients with no MR) were available as the patients were admitted to the Pediatric research ward at QECH only when they were clinically diagnosed with CM. However, efforts will be made to maintain the prevalence of MR and non-MR cases to avoid any bias in the dataset. The use of advanced classifier algorithms such as support vector machine or deep convolutional neural networks may also contribute in the performance improvement. Future development will be based on images prospectively acquired in both adult and pediatric patients across a spectrum of malaria transmission intensities and using a number of different image acquisition devices.

It is possible that transmission intensity of pediatric CM varies between geographical regions in sub-Saharan Africa. The dataset collection and relevant clinical studies for this research were conducted in malaria transmission settings in Malawi that are probably fairly representative of regions with prominent CM incidence in Africa^38^. Most of the research background, data, and clinical association established between malarial retinopathy and histopathologically confirmed cerebral malaria, also pertain to the similar geographical context. However, the proposed technology ought to help improve the accuracy of CM diagnosis as well as facilitate studies of clinical associations with malarial retinopathy, regardless of transmission intensity, since it provides an objective assessment that can be used in addition to indirect ophthalmoscopy. Nonetheless, we will continue with our efforts to test and validate the proposed system in the patient populations across the malaria transmission spectrum.

In summary, we have demonstrated a software based detection of MR lesions and a regression classifier that categorizes clinically defined CM in Malawian children into MR/no-MR, using retinal color images. The software algorithms perform with sufficient accuracy to enable a detection of MR with sufficient specificity for clinical and research use.

Apart from a clinical use, the automated MR detection software has potential in assisting CM researchers in both research and clinical settings. Currently, CM researchers have access only to a subjective observational data on MR lesion development and prevalence, and they can benefit from a quantified detection of MR lesions and relevant statistics, in several CM and MR research studies. This data may include, but not limited to, MR lesion location, quantity, size, color, special characteristics (e.g. if the hemorrhage is white-centered), lesion severity; the information that can be quantitatively correlated with clinical symptoms/parameters, medical observations, longitudinal health related changes, and risk of fatality. This imaging and image processing technology has potential to facilitate future clinico-pathological studies, not to mention studies of retinopathy in comatose children in general, by making retinal assessment much easier.

## Conclusions

We present the first fully automated software system for comprehensive detection of MR lesions and their statistically optimal combination to detect presence/absence of MR with high accuracy. The software is tuned to yield high specificity and positive predictive value, addressing the current clinical requirement to minimize over-diagnosis of CM. Our future efforts will focus on testing the software in a different age groups across the malaria transmission spectrum, and in refining the software algorithms for accuracy and optimized speed. In future, when integrated with a low-cost, portable retinal camera, the proposed software system may allow for the use in settings with limited access to ophthalmologists and ophthalmologic equipment.

## Additional Information

**How to cite this article**: Joshi, V. *et al*. Automated Detection of Malarial Retinopathy in Digital Fundus Images for Improved Diagnosis in Malawian Children with Clinically Defined Cerebral Malaria. *Sci. Rep.* **7**, 42703; doi: 10.1038/srep42703 (2017).

**Publisher's note:** Springer Nature remains neutral with regard to jurisdictional claims in published maps and institutional affiliations.

## Figures and Tables

**Table 1:** Performance for MR detection algorithms.

Algorithm Detection	AUC	Specificity	Sensitivity
MR detection	0.97	100%	95%
Hemorrhages	0.89	96%	73%
Vessel discoloration	0.85	100%	62%
Whitening	0.81	94%	65%
